# Evaluation of the Relationship between Impacted Maxillary Canine Teeth and Root Resorption in Adjacent Teeth: A Cross-Sectional Cone Beam Computed Tomography Study

**DOI:** 10.3390/diagnostics14141470

**Published:** 2024-07-09

**Authors:** Ahmet Aktı, Uğur Dolunay, Doğan Ilgaz Kaya, Gökhan Gürses, Doğucan Yeşil

**Affiliations:** 1 Department of Oral and Maxillofacial Surgery, Dentistry Faculty, Selçuk University, 42250 Konya, Turkey; ugurdolunayy@gmail.com (U.D.); gokhangurses.akademik@gmail.com (G.G.); dogucan_yesil@hotmail.com (D.Y.); 2 Department of Oral and Maxillofacial Surgery, Dentistry Faculty, Karamanoğlu Mehmetbey University, 70200 Karaman, Turkey; dt.doganilgaz@gmail.com

**Keywords:** impacted maxillary canine, root resorption, cone beam computed tomography

## Abstract

Background: This study aims to determine the position of impacted maxillary canines on cone beam computed tomography (CBCT) images, to determine the presence of resorption in adjacent teeth, and to investigate the position and type of resorption of impacted canines if resorption is present. Methods: Patients over 14 years of age with maxillary canine teeth who had CBCT images taken for any dental reason were included in the study. Resorption of teeth adjacent to maxillary canines was analyzed according to transversal, vertical, and buccopalatinal positions on the CBCT. The study evaluated 162 maxillary canine teeth on tomography images of 134 patients. Results: Of the affected adjacent teeth, 32.1% of the central incisor, 58.0% of the lateral incisor, and 19.1% of the first premolars showed mild-to-severe resorption. The relationships between transversal position and resorption in the central and lateral incisor, vertical position and resorption in the central incisor and buccopalatinal position and resorption in the first premolars were found to be significant. Maxillary canine teeth can cause mild-to-severe resorption of adjacent teeth, especially lateral incisors. Conclusion: For this reason, we think that a detailed examination with CBCT is essential in the early diagnosis of resorption of adjacent teeth.

## 1. Introduction

Impaction is defined as a failure of tooth eruption within its normal period of growth at its appropriate site in the dental arch. Impacted maxillary canine teeth are among the most commonly impacted teeth in the jaw. The prevalence of impacted maxillary canines is between 1 and 3%, and they are the second most frequently impacted teeth after mandibular third molars [1,2,3,4]. Impacted canines are considered a multifactorial condition, with genetic factors playing an important role, both locally and systemically. The canines will not erupt in the correct position if they deviate from the standard eruption path. This may be due to a lack of space for the teeth to erupt or a congenital absence of lateral incisors. The latter reason is explained by the orientation theory, which suggests that the lateral incisor guides the canine eruption [5]. Other local factors that play a critical role in impacted canines include differences in arch length and tooth size, failure of root resorption in the deciduous canine, early loss of the deciduous canine or permanent lateral incisor, dilaceration of the root, and variations in the eruption time of the canine and permanent lateral incisor [6,7,8]. Maxillary canines are among the most esthetically and functionally essential teeth. However, their treatment is difficult and time-consuming when they remain impacted. The location and position of impacted canines vary greatly and may cause root resorption and cystic degeneration in adjacent teeth. For this reason, it is crucial to determine the location of the impacted canines, their relationship with the adjacent teeth, and whether they cause any root resorption or cystic degeneration with the use of detailed radiographic imaging [9,10]. Although many conventional radiographs can be used for this purpose, cone beam computed tomography (CBCT) provides the most detailed information [11].

Two-dimensional radiography (2D) methods help determine the position of impacted teeth. However, they cannot provide clear information about the presence, degree, and cystic degeneration of root resorption in adjacent teeth due to their overlap with adjacent tooth roots. The importance of three-dimensional radiography (3D) methods, especially CBCT, emerges as a consequence [10]. CBCT is frequently used today due to its ability to eliminate superimposed structures, provide excellent tissue contrast, and generate precise 3D images. In many cases, it can even change the planning arrived at with the use of conventional radiographs [7].

This study aims to determine the position of impacted maxillary canines on CBCT images, determine the presence of resorption in the adjacent teeth, and, if resorption is present, investigate the position of the impacted canines in this situation and how they lead to resorption. This study aims to contribute to the accurate evaluation of impacted maxillary canines, which plays an essential role in surgical and orthodontic treatment planning in clinical applications.

## 2. Materials and Methods

This study was conducted following the principles of the Declaration of Helsinki and was approved by the Selcuk University Non-Interventional Ethics Committee (decision 2024/10). The tomography images of 193 patients over 14 years of age with impacted maxillary canine teeth who had previously undergone CBCT for any dental reason were analyzed.

The tomography images of patients who had or were undergoing orthodontic treatment, who were missing any of the teeth (central, lateral, and first premolar) adjacent to the impacted canine, who had cleft lip and palate, who had a history of maxillofacial pathology or fracture adjacent to the impacted canine, who had excessive crown destruction in any of the teeth adjacent to the impacted canine, and who had poor image quality were excluded from the study. A total of 134 patients (91 females and 43 males) were included in the study.

These images were obtained using the same CBCT device (Instrumentarium Dental, Palo DEx Group Oy Nahkelantie 160 FI-04300, Tuusula, Finland). The radiographic parameters were 89 kVp and 4–12 mA. During the analysis process, special attention was paid to the complete visualization of the impacted teeth and their adjacent teeth. Images from all field-of-view (FOV) areas were included in the study to examine root resorption in adjacent teeth.

An oral and maxillofacial surgeon with four years of experience in CBCT examination in axial, coronal, and sagittal sections performed all measurements and evaluations.

First, the transversal position of the impacted maxillary canine cusp apex on CBCT was determined according to the position of the adjacent tooth. This position was evaluated in six different regions based on the revised version of the modified transversal classification proposed by Leonardi et al. and Kök and Aşık [12,13].

**Figure 1:**
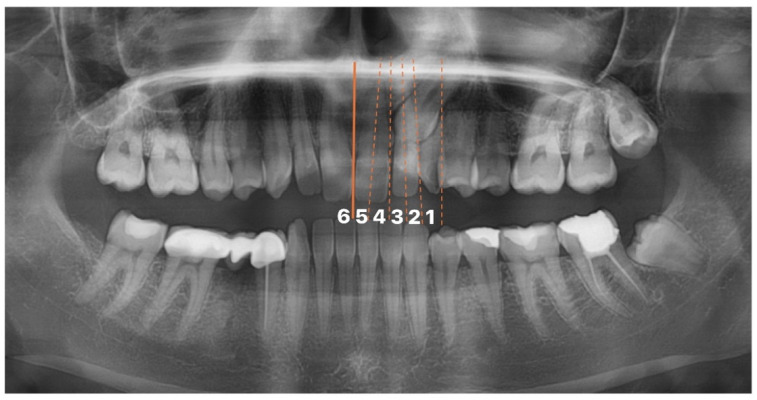
Classification of impacted maxillary canines according to their transversal position (1—Canine zone, 2—Lateral distal zone, 3—Lateral mesial zone, 4—Central distal zone, 5—Central mesial region, 6—Opposite side).

The classification refers to the following regions: 1. Canine Zone, the area distal to the lateral incisor where the deciduous canine tooth is located, and where the permanent canine is expected to be located, 2. Lateral Distal Zone, the area between the line drawn from the distal contact point of the lateral incisor and the long axis of the lateral incisor, 3. Lateral Mesial Zone, the area between the long axis of the lateral incisor and the line drawn from the mesial contact point of the lateral incisor, 4. Central Distal Zone, the area between the line drawn from the distal contact point of the central incisor and the long axis of the central incisor, 5. Central Mesial Region, the area between the long axis of the central incisor and the line drawn from the mesial contact point, and 6. Opposite Side, this refers to a tooth that has crossed the midline drawn between two central incisors and is located in the other segment. The classification is shown in Figure 1.

**Figure 2:**
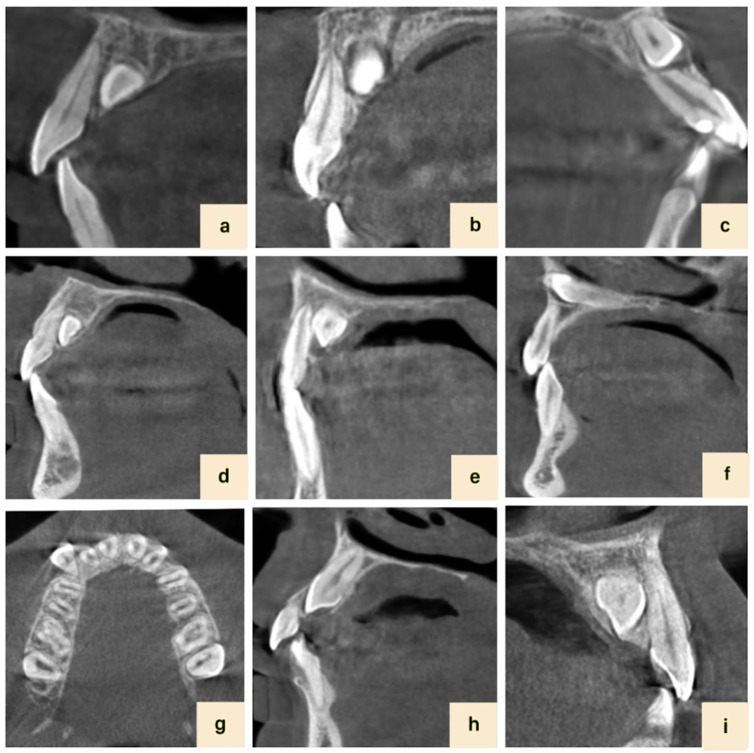
Position and resorption status of impacted canine teeth: (**a**) mildly severe resorption, (**b**) moderate resorption, (**c**) severe resorption, (**d**) coronal position, (**e**) middle position, (**f**) apical position, (**g**) buccal position, (**h**) mid-alveolar position, and (**i**) palatal position.

The vertical position of the crown of the impacted canine tooth was then determined as “apical, middle, coronal” in terms of the root of the associated tooth as shown in Figure 2 [14]. The labio-palatal position of the impacted canine tooth was determined as “labial, mid-alveolar, palatal” in cross-sectional CBCT images (see Figure 2).

The degree of root resorption is classified into four groups according to the classification proposed by Ericson and Kurol:

Grade 0: no resorption;

Grade 1: mild resorption (extending to the dentin midline);

Grade 2: moderate severity of resorption (exceeding the dentin midline but not reaching the pulp);

Grade 3: severe resorption (reaching the pulp) [10,15].

### Statistical Analysis

In the analysis of demographic data, descriptive statistics were first obtained using basic statistical methods. In this context, frequency analysis was used to examine the data distribution and report the observed frequency of each category. In addition, mean and standard deviation measurements were calculated to understand the central tendency and distribution of the continuous variables of the data. The Kolmogorov–Smirnov test assessed the conformity of the data to normal distribution. Chi-square cross-tabulation analysis was performed for two-category relationship analysis. All analyses were performed using the SPSS 22 package program (SPSS Inc., Chicago, IL, USA).

## 3. Results

The study sample consisted of the CBCT images of 134 patients who met the inclusion criteria. Patients who had, or were undergoing, orthodontic treatment (*n*: 7), missing teeth (central, lateral, and first premolars) adjacent to the impacted canine (*n*: 26), cleft lip and palate (*n*: 8), a history of maxillofacial pathology or fracture adjacent to the impacted canine (*n*: 8), excessive crown destruction in any of the teeth adjacent to the impacted canine (*n*: 6), and poor image quality (*n*: 4) were excluded. Within the scope of the study, 162 impacted canines were evaluated.

The mean age range of the patients who participated in the study was between 14 and 69 with a mean of 26.7 ± 13.1. Of the participants, 91 (67.9%) were female and 43 (32.1%) were male. Regarding the status of impacted canines, 28 (20.9%) were bilateral, 42 (31.3%) were right unilateral, and 64 (47.8%) were left unilateral. In general, 70 (43.2%) impacted canines were on the right, and 92 (56.8%) were on the left. Chi-square analyses, performed to reveal the correlational difference between the resorption of adjacent teeth in terms of right and left statuses, are given in Table 1.

When the resorption rates in the adjacent teeth were analyzed, 10 (6.2%) central incisors, 30 (18.5%) lateral incisors, and 8 (4.9%) first premolars had severe resorption.

Regarding the transversal position of the impacted canines, 27 (16.7%) were in position 1, 34 (21.0%) in position 2, 28 (17.3%) in position 3, 33 (20.4%) in position 4, 27 (16.7%) in position 5, and 13 (8.0%) in position 6. Chi-square analyses, performed to reveal the correlational differences between the resorption of adjacent teeth in terms of their transversal position, are given in Table 2.

In terms of the vertical position, 76 (46.9%) were coronal, 69 (42.6%) intermediate, and 17 (10.5%) apical. Table 3 shows Chi-square analyses performed to reveal the correlational difference between the vertical position and the resorption of adjacent teeth.

In terms of the buccopalatal position, 25 (15.4%) were buccal, 42 (25.9%) mid-alveolar, and 95 (58.6%) palatal. Table 4 provides chi-square analyses performed to reveal the correlational difference between the buccopalatal position and the resorption of adjacent teeth.

The relationship between the resorption of central incisors, according to whether the impacted maxillary canine was on the right or left side, was examined, and the *p*-value obtained as a result of the chi-square analysis was found to be 0.009, which indicates a statistically significant relationship (*p* < 0.05).

In right-sided impacted canines, resorption was not observed in 67.1% of the central incisors. In those where resorption was observed, signs of moderate resorption were mainly observed. However, in the case of left-sided impacted canines, the resorption of the central incisor was usually absent or mild.

The *p*-value obtained from the chi-square analysis to examine the correlational difference between lateral resorption according to the right–left status was 0.629, which does not indicate a statistically significant relationship (*p* > 0.05). These results suggest that there is mostly no resorption, irrespective of the left and right statuses.

Similarly, the *p*-value obtained from the chi-square analysis to examine the correlational difference between resorption in the first premolars in terms of the right–left status was 0.727, which does not indicate a statistically significant relationship (*p* > 0.05). These results show that there is mostly no resorption, regardless of the left and right situations.

Chi-square analysis was used to analyze the relationship between the transversal position and central incisor resorption. According to the analysis results, the *p*-value obtained was *p* < 0.001, which indicates a significant relationship (*p* < 0.05). No signs of resorption were consistently observed in the central incisor of individuals with transversal positions 1, 2, and 3 (resorption status = 0). Likewise, individuals with the transversal position 4 usually showed no signs of resorption in the central incisor (resorption status = 0). However, in individuals with transversal position 5, the resorption status of the central incisor was usually of grade 1. In addition, in individuals with a transversal position of 6, the resorption status of the central incisor was usually grade 2. These findings reveal a significant relationship between the transversal position and central incisor resorption. In particular, it was observed that resorption in the central incisor was more pronounced in the case of individuals with certain transversal positions.

As a result of the chi-square analysis, the relationship between the transversal position and lateral incisor resorption was analyzed. Based on the results obtained, the *p*-value was found to be *p* < 0.001, which indicates a significant relationship (*p* < 0.05). Based on the analysis results, no signs of resorption were generally observed in the lateral incisors of individuals with transversal positions 1, 3, 4, and 6 (resorption status = 0). However, in individuals with transversal position 2, the resorption status of the lateral incisor was generally determined as grade 2. In addition, in individuals with transversal position 5, the resorption status of the lateral incisor was generally grade 3. These findings reveal a significant relationship between transversal position and lateral incisor resorption. In particular, it was observed that resorption in the lateral incisor was more pronounced in individuals with certain transverse positions.

The results of the chi-square analysis were considered by examining the relationship between the transversal position and the first premolar tooth. Based on the analysis results, the *p*-value was 0.071, which does not indicate a statistically significant relationship (*p* > 0.05). Based on these findings, the transversal position has no statistically significant effect on the resorption status of the first premolars. Regardless of the transversal position, the resorption status of the first premolars was mostly grade 0. These results suggest that the resorption of premolars may be due to different factors rather than the transverse position.

The *p*-value obtained from the chi-square analysis with regard to examining the correlational difference between the vertical position and central incisor resorption was 0.002, indicating a statistically significant relationship (*p* < 0.05). Based on the results of the analysis, no signs of resorption were generally observed in the central incisor of individuals with coronal vertical positions. On the other hand, in individuals with mid-alveolar vertical positions, the resorption of central incisors was generally absent or grade 2. Furthermore, in individuals with apical vertical positions, the resorption of the central incisor was generally absent or grade 3.

The *p*-value obtained from the chi-square analysis with regard to examining the relationship between the vertical position and lateral incisor resorption was 0.099, which does not indicate a statistically significant relationship (*p* > 0.05). Similarly, the *p*-value obtained from the chi-square analysis with regard to examining the relationship between the vertical position and first premolar tooth resorption was 0.073, which does not indicate a statistically significant relationship (*p* > 0.05).

Based on the analysis results, central incisor resorption was generally not detected in individuals with buccal and mid-alveolar buccopalatal positions. However, in individuals with palatal buccopalatal positions, the resorption of the central incisor was generally not seen or detected as grade 1. The *p*-value obtained from the chi-square analysis to examine the relationship between buccopalatal position and lateral incisor resorption was 0.939, indicating no statistically significant relationship (*p* > 0.05).

Similarly, the *p*-value obtained from the chi-square analysis to examine the relationship between the buccopalatal position and first premolar tooth resorption was 0.001, indicating a statistically significant relationship (*p* < 0.05). Based on these results, in individuals with buccal buccopalatal position, the resorption of the first premolars was generally not seen or was seen as grade 2. In individuals with the mid-alveolar buccopalatal position, first premolars were typically not resorbed or grade 1. In individuals with the palatal buccopalatal position, first premolars were generally not resorbed.

## 4. Discussion

In individuals older than ten years of age with maxillary canines that have not yet erupted, detailed radiographic examination is critical to prevent the possible root resorption and pathological conditions in teeth adjacent to impacted canines. Two-dimensional radiographs are usually used for evaluation and treatment planning in the first stage. However, two-dimensional radiographic imaging techniques are inadequate when it comes to evaluating root resorption caused by impacted maxillary canines because they do not allow the detailed examination of resorption and pathologic conditions. Therefore, 3D imaging techniques are often preferred for accurate diagnosis when it comes to determining the position and root resorption of impacted canines [16]. Compared to conventional computed tomography (CT), CBCT devices have been reported to be more widely used due to their lower radiation output and lower cost [17,18]. In the literature, there are several studies in which impacted maxillary canines were examined using 3D imaging techniques. In these studies, the positions of impacted maxillary canines and root resorption in adjacent incisors were evaluated in terms of the images obtained in these studies [9,18,19]. In our study, we preferred to use CBCT to perform a detailed analysis. In addition to the studies in the literature, in our research, we also examined root resorption in the first premolars.

For this purpose, the transversal, vertical, and buccopalatinal positions of the teeth were evaluated, and whether they resorbed the adjacent central, lateral, and first premolar teeth was examined.

The literature shows that the prevalence of impacted canines ranks second after mandibular third molars and varies between 1 and 3% [3,4]. Studies have shown that impacted maxillary canines are frequently located in the palatinal region [2,9,20,21]. In our study, the impacted maxillary canines were frequently located on the palatal side, in line with the findings in the literature. In addition, it has been reported that impacted maxillary canines are more common in females than males [3,21,22,23]. In this study, we observed a female majority with an average ratio of 2/1.

Impacted maxillary canines can cause the root resorption of adjacent teeth with different severities. Many scoring methods are used in the literature to determine root resorption and its degree. Ericson and Kurol scored resorption into four categories, i.e., no, mild, moderate, and severe resorption [10,15]. Since this grading system has been used in many studies evaluating the amount of resorption resulting from the ectopic eruption of impacted maxillary canines, we used this grading system in our study [18,24].

In studies in the literature, it has been reported that the rates of incisor resorption resulting from the ectopic eruption of the maxillary canine vary between 4.9 and 38% [9,18,25,26]. In their CBCT study, Liu et al. examined the resorption of adjacent incisors associated with 210 impacted maxillary canine teeth. They observed resorption in 105 teeth, 49 (23.4%) of which were central and 56 (27.2%) lateral incisors [18]. Preda et al. examined the resorption of adjacent incisors associated with 29 impacted maxillary canine teeth and observed resorption in 8 teeth, 2 (3.6%) of which were central, and 6 (10.8%) of which were lateral incisors [2]. In their CBCT study, Walker et al. examined the resorption of adjacent incisors associated with 27 impacted maxillary canine teeth. They reported resorption in a total of 21 teeth, of which 3 (11.1%) were central and 18 (66.6%) were lateral incisors [3]. In their CBCT study, Doğramaci et al. examined the resorption in the incisors and all teeth associated with 110 impacted maxillary canine teeth. They reported that they saw resorption in 120 teeth, including 28 central incisors (23.3%), 77 lateral incisors (64.1%), 14 first premolars (11.6%), and 1 first molar (0.8%) [27]. Similarly, in their CT study, Cernochova et al. examined the resorption of 334 impacted maxillary canine and adjacent teeth. They reported severe resorption findings in 63 teeth, 7 (2.1%) of which were central incisors, 40 (12.6%) lateral incisors, and 16 (4.8%) first premolars [28]. In our study, we observed resorption in 52 (32.1%) central incisors, 94 (58%) lateral incisors, and 31 (19.1%) first premolar teeth associated with 134 impacted maxillary canines. Furthermore, 10 (6.2%) of the central incisors had severe resorption, 30 (18.5%) of the lateral incisors had severe resorption, and 8 (4.9%) of the first premolar teeth had severe resorption. According to the results, it is understood that, unlike CT studies, the resorption findings in our study with the use of CBCT were more clearly seen and reflected in the rates, in parallel with similar CBCT studies.

Regardless of the imaging technique used, the teeth most affected by impacted canines were the lateral incisors, with a rate of 58% in our study as in other studies. In addition, we observed 19.1% of resorption in the first premolars, which is usually ignored in different studies.

The resorption of impacted maxillary canines and associated teeth can be evaluated in terms of the transversal position. In the literature, Jung et al. [29] and Ngo et al. [20] used the same classification and assessed five groups without including the teeth beyond the midline. In addition to this classification, Kök and Aşık [13] created six groups by considering the embedded canines that cross the midline. This study preferred Kök and Aşık’s classification since impacted canines crossed the midline.

In studies examining the relationship between the transversal position and resorption, Ericson and Kurol [10] reported that impacted maxillary canines in zones 3 and 4 cause the resorption of lateral incisors. Jung et al. [29] and Ngo et al. [20] reported an increased risk of resorption of maxillary incisors in zones 4 and 5. Chaushu et al. [30] also described how the severe resorption of incisors was predominant in zones 5 and 4, respectively. In the present study, lateral incisor resorption was primarily observed in zones 3, 4, and 5, while incisor resorption was primarily observed in zones 4 and 5, in line with the literature.

The vertical position is another parameter associated with the resorption of impacted maxillary canines. Chaushu et al. [30] and Schroder et al. [31] found that impacted maxillary canines were mainly positioned in the middle region (62.3%) in incisors with resorption. Lai et al. [32] also stated that impacted canines in coronal or apical positions caused less resorption. Our study observed that resorption occurred mainly in the middle position in the case of central incisors and mostly in the coronal position in lateral and first premolars.

The strengths of our study are that it was performed with the use of CBCT, a large number of cases were screened, and the resorption status of premolars was also examined. However, our study is a retrospective one, and analyses were performed on the available CBCT data. Evaluating a larger sample group to obtain more detailed information would increase the reliability of the study. There is a need for similar detailed studies on the subject in different populations.

## 5. Conclusions

Impacted maxillary canines tend to cause significant resorption of adjacent teeth, especially lateral incisors. CBCT shows the resorption of impacted maxillary canines on adjacent teeth in detail. Therefore, the CBCT evaluation of impacted maxillary canines is crucial for accurate diagnosis and treatment planning. Early clinical follow-up and CBCT evaluations would prevent possible resorption and the problems that may occur in adjacent teeth by taking precautions aimed at preventing canine teeth from becoming impacted.

## Figures and Tables

**Table 1:** Examination of resorptions according to the right and left sides.

	Right			Left		
	Central	Lateral	First Premolar	Central	Lateral	First Premolar
No resorption	47 (67.1%)	32 (45.7%)	57 (81.4%)	63 (68.5%)	36 (39.1%)	74 (80.4%)
Mild	5 (7.1%)	9 (12.9%)	7 (10.0%)	18 (19.6%)	18 (19.6%)	8 (8.7%)
Moderate	14 (20.0%)	15 (21.4%)	4 (5.7%)	5 (5.4%)	22 (23.9%)	4 (4.3%)
Severe	4 (5.7%)	14 (20.0%)	2 (2.9%)	6 (6.5%)	16 (17.4%)	6 (6.5%)
Total	70 (100%)	70 (100%)	70 (100%)	92 (100%)	92 (100%)	92 (100%)

**Table 2:** Examination of resorption in adjacent teeth according to their transversal position.

	Central						Lateral							First Premolar				
			Resorption *n* (%)															
TK	0	1	2	3	Total	*p*	0	1	2	3	Total	*p*	0	1	2	3	Total	*p*
1	27 (100)	0 (0.0)	0 (0.0)	0 (0.0)	27 (100)	<0.001	24 (88.9)	1 (3.7)	1 (3.7)	1 (3.7)	27 (100)	<0.001	18 (66.7)	5 (18.5)	2 (7.4)	2 (7.4)	27 (100)	0.071
2	34 (100)	0 (0.0)	0 (0.0)	0 (0.0)	34 (100)		10 (29.4)	5 (14.7)	14 (41.2)	5 (14.7)	34 (100)		23 (67.6)	4 (11.8)	5 (14.7)	2 (5.9)	34 (100)	
3	28 (100)	0 (0.0)	0 (0.0)	0 (0.0)	28 (100)		8 (28.6)	7 (25.0)	8 (28.6)	5 (17.9)	28 (100)		24 (85.7)	3 (10.7)	1 (3.6)	0 (0.00)	28 (100)	
4	14 (42.4)	9 (27.3)	5 (15.2)	5 (15.2)	33 (100)		11 (33. 3)	9 (27.3)	8 (24.2)	5 (15.2)	33 (100)		27 (81.8)	3 (9.1)	0 (0.0)	3 (9.1)	33 (100)	
5	6 (22.2)	10 (37.0)	8 (29.6)	3 (29.6)	27 (100)		7 (25.9)	3 (11.1)	5 (18.5)	12 (44.4)	27 (100)		26 (96.3)	0 (0.0)	0 (0.0)	1 (3.7)	27 (100)	
6	1 (7.7)	4 (30.8)	6 (46.2)	2 (15.4)	13 (100)		8 (61.5)	2 (15.4)	1 (7.7)	2 (16.4)	13 (100)		13 (100)	0 (0.0)	0 (0.0)	0 (0.0)	13 (100)	
T	110 (67.9)	23 (14.2)	19 (11.7)	10 (6.2)	162 (100)		68 (42. 0)	27 (16.7)	37 (22.8)	30 (18.5)	162 (100)		131 (80.9)	15 (9.3)	8 (4.9)	8 (4.9)	162 (100)	

**Table 3:** Examination of resorption in adjacent teeth according to their vertical position.

	Central						Lateral						First Premolar					
				Resorption *n* (%)														
VK	0	1	2	3	Total	*p*	0	1	2	3	Total	*p*	0	1	2	3	Total	*p*
K	60 (78.9)	9 (11.8)	5 (6.6)	2 (2.6)	76 (100)	0.002	35 (46.1)	16 (21.1)	17 (22.4)	8 (10.5)	76 (100)	0.099	60 (78.9)	11 (14.5)	3 (3.9)	2 (2.6)	76 (100)	0.073
O	39 (56.5)	12 (17.4)	14 (20.3)	4 (5.8)	69 (100)		28 (40.6)	9 (13.0)	17 (24.6)	15 (21.7)	69 (100)		58 (84.1)	1 (1.4)	5 (7.2)	5 (7.2)	69 (100)	
A	11 (64.7)	2 (11. 8)	0 (0.0)	4 (23.5)	17 (100)		5 (29.4)	2 (11.8)	3 (17.6)	7 (41.2)	17 (100)		13 (76.5)	3 (17.6)	0 (0)	1 (5.9)	17 (100)	
T	110 (67.9)	23 (14.2)	19 (11.7)	10 (6.2)	162 (100)		68 (42.0)	27 (16.7)	37 (22.8)	30 (18.5)	162 (100)		131 (100)	15 (9.3)	8 (4.9)	8 (4.9)	162 (100)	

**Table 4:** Evaluation of resorptions according to the buccopalatal position.

		Central					Lateral						First Premolar					
			Resorption *n* (%)															
BPK	0	1	2	3	Total	*p*	0	1	2	3	Total	*p*	0	1	2	3	Total	*p*
B	20 (80.0)	2 (8.0)	1 (4.0)	2 (8.0)	25 (100)	0.001	11 (44)	5 (20.0)	4 (16.0)	5 (20.0)	25 (100)	0.939	17 (68.0)	3 (12.0)	5 (20.0)	0 (0.0)	25 (100)	0.001
M	38 (90.5)	1 (2.4)	1 (2.4)	2 (4.8)	42 (100)		20 (47.6)	6 (14.3)	9 (21.4)	7 (16.7)	42 (100)		30 (71.4)	7 (16.7)	1 (2.4)	4 (9.5)	42 (100)	
*p*	52 (54.7)	20 (21.1)	17 (17.9)	6 (6.3)	95 (100)		37 (38.9)	16 (16.8)	24 (25.3)	18 (18.9)	95 (100)		84 (88.4)	5 (5.3)	2 (2.1)	4 (4.2)	95 (100)	
T	110 (67.9)	23 (14.2)	19 (11.7)	10 (6.2)	162 (100)		68 (42.0)	27 (16.7)	37 (22.8)	30 (18.5)	162 (100)		131 (80.9)	15 (19.3)	8 (4.9)	8 (4.9)	162 (100)	

## Data Availability

The dataset is available upon request from the authors.
